# The Suppression of Symptoms

**Published:** 1893-09

**Authors:** 


THE SUPPRESSION OF SYMPTOMS by massive doses of drugs or by topical applications would seem to be so familiar that no physician could be found who would treat in that way. Yet most old-school practitioners and many homœopathists resort to such measures, and when the results arise deny the cause. The case related by Dr. Wesselhœft, on page 465, is a very striking and rare illustration of the principle of metastasis after suppression. The editor has seen a case of violent headache caused by suppressing the intense itching, burning, and stinging of an eruption on the leg, by applications of salt water.

EDITOR.

